# Mental Disorders Are Associated With Leukocytes Telomere Shortening Among People Who Inject Drugs

**DOI:** 10.3389/fpsyt.2022.846844

**Published:** 2022-06-17

**Authors:** Mélusine Durand, Nicolas Nagot, Laurent Michel, Sao Mai Le, Huong Thi Duong, Roselyne Vallo, Amélie Vizeneux, Delphine Rapoud, Hoang Thi Giang, Catherine Quillet, Nham Thi Tuyet Thanh, Khuat Thi Hai Oanh, Vu Hai Vinh, Jonathan Feelemyer, Philippe Vande Perre, Khue Pham Minh, Didier Laureillard, Don Des Jarlais, Jean-Pierre Molès

**Affiliations:** ^1^Pathogenesis and Control of Chronic and Emerging Infections, University of Montpellier, INSERM, EFS, University of Antilles, Montpellier, France; ^2^Pierre Nicole Center, CESP UMR 1018, Paris-Saclay University, Paris, France; ^3^Faculty of Public Health, Hai Phong University of Medicine and Pharmacy, Hai Phong, Vietnam; ^4^Supporting Community Development Initiatives, Hanoi, Vietnam; ^5^Infectious and Tropical Diseases Department, Viet Tiep Hospital, Hai Phong, Vietnam; ^6^School of Global Public Health, New York University, New York, NY, United States; ^7^Infectious and Tropical Diseases Department, Caremeau University Hospital, Nîmes, France

**Keywords:** drug use, depression, psychotic syndrome, telomere shortening, mitochondrial DNA, hepatitis C virus, aging

## Abstract

Premature biological aging, assessed by shorter telomere length (TL) and mitochondrial DNA (mtDNA) alterations, has been reported among people with major depressive disorders or psychotic disorders. However, these markers have never been assessed together among people who inject drugs (PWIDs), although mental disorders are highly prevalent in this population, which, in addition, is subject to other aggravating exposures. Diagnosis of mental disorders was performed by a psychiatrist using the Mini International Neuropsychiatric Interview test among active PWIDs in Haiphong, Vietnam. mtDNA copy number (MCN), mtDNA deletion, and TL were assessed by quantitative PCR and compared to those without any mental disorder. We next performed a multivariate analysis to identify risk factors associated with being diagnosed with a major depressive episode (MDE) or a psychotic syndrome (PS). In total, 130 and 136 PWIDs with and without psychiatric conditions were analyzed. Among PWIDs with mental disorders, 110 and 74 were diagnosed with MDE and PS, respectively. TL attrition was significantly associated with hepatitis C virus-infected PWIDs with MDE or PS (adjusted odds ratio [OR]: 0.53 [0.36; 0.80] and 0.59 [0.39; 0.88], respectively). TL attrition was even stronger when PWIDs cumulated at least two episodes of major depressive disorders. On the other hand, no difference was observed in mtDNA alterations between groups. The telomeric age difference with drug users without a diagnosis of psychiatric condition was estimated during 4.2–12.8 years according to the number of MDEs, making this group more prone to age-related diseases.

## Introduction

Psychiatric conditions among people who inject drugs (PWIDs) are of great concern. Their prevalence is extremely high with an estimated 40% of PWIDs being affected, making psychiatric disorders one of the most frequent co-morbidities associated with substance use disorder (1–3). Moreover, the type of substance is also an important factor to take into consideration, psychosis being more frequent among methamphetamine users (4–7), while heroin users are more prone to depression (8, 9).

Both mental disorders and substance use have been identified as risk factors for premature morbidities and mortalities, increased psychosocial distress, and an increased risk of blood-borne viral acquisition and transmission (10). Among the comorbidities, cardiovascular impairment, insulin resistance, and neurodegenerative disorders (11–13) are frequent. Depressive disorders have been associated with higher levels of inflammatory and metabolic markers, which also increase the risk of developing cardiovascular diseases and type II diabetes (14). Schizophrenia has also been associated with an increased risk of cardiovascular diseases, diabetes, and high blood pressure (15). Meanwhile, methamphetamine use has been associated with increased risks for cardiovascular pathologies and for the development of Alzheimer’s or Parkinson’s diseases (16–18).

Biomarkers of cellular aging have been associated with many of these conditions. In some situations, they constitute early markers of disease development, enabling early interventions to prevent their progression. Markers of premature aging include mitochondrial DNA (mtDNA) integrity and telomere length (TL). Mitochondria are essential for adenosine triphosphate production, which provides the necessary energy for cell functions. They have their own DNA, a small and circular molecule of 16,569 bp coding for 13 proteins of the respiratory chain. A cell contains hundreds to thousands of mitochondria, which in turn contain 1–10 mtDNA molecules; the number of molecules varies depending on cell type and energy requirement. MtDNA is highly susceptible to genetic damages mainly because of its proximity to the respiratory chain, which produces oxygen radicals, nucleotide repair mechanisms less effective than the nuclear ones, and the absence of protective histone scaffolds. Cell response to mitochondrial injuries is to increase the number of mtDNA per cell in order to compensate for the mutated ones. Up to a certain threshold, altered mitochondria can also be eliminated through mitophagy, but a clonal expansion of the mutated molecule can be overtaken that results in a mitochondrial function that is no longer sustained (19). Quantification of mitochondrial copy number content (MCN) and its subsequent decrease and the rates of mtDNA mutations and deletions (MDD) have been proposed as a proxy for mitochondrial dysfunction and markers of cellular aging (20, 21). Similarly, TL attrition was identified as a marker of premature aging. Each telomere consists of 3–20 kb of a sequence of six repeated nucleotides – TTAGGG –, positioned at the ends of chromosomes, protecting them from degradation. They are synthesized by the telomerase, an RNA-dependent DNA polymerase, found in the stem and progenitor cells but missing in somatic cells, with the exception given to immature lymphocytes. Physiologically, telomeres shorten with age, but this process can be accelerated by individual and environmental factors.

These markers of premature cellular aging have been extensively studied among patients with psychiatric conditions. Among subjects with depressive symptoms, MCNs in blood have yielded discordant results: reports found it increased (22–24), decreased (25, 26), or without any change (27, 28) when compared to healthy subjects. Leukocyte TL, however, was almost unanimously found to be shortened among depressed subjects (22, 28–31), while this association is still discussed among people with psychotic disorders (32). Among drug users, decreases in MCN and increases in mtDNA damage have been reported among opiate-addicted patients (33), while methamphetamine has been shown to induce mitochondrial damages *in vitro* (34, 35). Moreover, 4,977-bp common deletion, which is highly associated with aging, was never investigated in these populations (36). Finally, TL has been shown to be shortened in leucocytes of drug-addicted subjects (37). Nevertheless, a comprehensive evaluation of three age-related biomarkers that include MCN, MDD, and TL, has never been studied, to our knowledge, among a population with substance use disorders and co-occurring mental illness.

In a recent study among injecting heroin users in Vietnam, we reported that one-third of them were suffering from psychiatric conditions, with a current major depressive episode (MDE) and psychotic syndromes (PSs) accounting for 21.3 and 15.1%, respectively, (38). Herein, we aimed to characterize markers of premature cellular aging among PWIDs with psychiatric disorders by quantifying MCN, MDD, and TL on whole blood DNA extracts.

## Materials and Methods

### Study Design and Study Population

We designed a cross-sectional observational study, between August 2018 and April 2019, among the participants of the DRIVE project (NTC03526939). This project had the primary objective to evaluate the impact of a community-based intervention for HIV screening and treatment among PWIDs to control HIV transmission in Hai Phong, Vietnam and consisted of 4 annual respondent-driven sampling surveys and biannual visits for those enrolled in the cohorts (39). PWIDs were eligible if they were (i) older than 18 years old, (ii) able to provide a signed informed consent, and (iii) actively injecting drugs as assessed by visible marks of injection and a urinary test positive for heroin and/or methamphetamine. In a face-to-face interview, community workers gathered information on socio-demographic data and drug use. Nurses provided HIV and hepatitis C virus (HCV) tests. During the biannual visit, participants had a consultation with a psychiatrist who diagnosed potential mental health problems, using the Mini International Neuropsychiatric Interview (MINI), a structured psychiatric interview based on the Diagnostic and Statistical Manual of Mental Disorders, Fourth Edition (DSM-IV) criteria (40). If necessary, psychiatric treatments were initiated. The MINI test was used to diagnose current and/or lifetime MDE, current and/or lifetime PS, and to evaluate suicidal risk.

For the present study, we included PWIDs who had banked blood samples and who had a psychiatric interview (Supplementary Figure 1). PWIDs were next divided into two groups: a group of PWIDs with psychiatric conditions, defined by subjects with at least one psychiatric condition diagnosed among either MDE or PS, and a control group of PWIDs without either of these conditions. The population with MDE included both those with a current or a lifetime MDE. Similarly, the population with PS included both those with a current or a lifetime PS.

The present study complies with the Declaration of Helsinki and was approved by the Institutional Review Board of the Haiphong University of Medicine and Pharmacy, Vietnam (#01/HPUMPRB) and Mount Sinai Beth Israel hospital, New York, United States.

### Blood Collection and DNA Extraction

In total, 80 μl of whole blood was collected on dried blood spot cards (DBS, Whatman™ 903, GE Healthcare Bio Sciences Corp.), dried, and stored at −80°C. DNA was extracted after blood elution using a QIAamp DNA Mini Kit, following the instructions of the manufacturer, and stored at −80°C until further process.

### Mitochondrial DNA Copy Number and Mitochondrial DNA Deletion Assays

Mitochondrial DNA copy number was assessed by a commercial assay (QuickScanTM Mitox Kit, Primagen, Amsterdam, Netherlands) targeting rRNA 16S in the mtDNA genome and snRNP U1A in the genomic DNA. Briefly, 2.5 μl of the sample extract was added to a mixture of primers (Supplementary Table 1) at a final concentration of 500 nM and LC480^®^ SYBR Green I Master Mix 2X (Roche, Germany) for a final volume of 25 μl. Genomic and mitochondrial targets were amplified separately in LightCycler^®^ 480 Instrument (Roche, Germany). The amplification program consisted of 5 min of incubation at 95°C followed by 45 cycles of 10 s at 95°C, 20 s at 60°C, and 20 s at 72°C. MCN was obtained by reporting the Ct of each target on an external standard curve, made with serial dilution of calibrator consisting of plasmids with rRNA 16S and snRNP U1A inserts. In each plate, one calibrator, one control, and one no template control were added. The plate was valid if the calibrator and control were within the expected range and no amplification occurred in the no template well. The sample was valid if the Ct value did not exceed 30. MCN was expressed in copies of mtDNA/cell (c/cell), assuming each cell has two copies of genomic DNA.

Mitochondrial DNA deletion was quantified by quantitative PCR (qPCR) that targeted two regions of the mtDNA, the ND1 gene and the ND4 gene (primers described in Supplementary Table 1); in the latter, more than 85% of the deletions reported so far were found (41). The reaction mixture consisted of 5 μl of sample extract, forward primers, and probes at a final concentration of 100 nM, reverse primers at a concentration of 250 nM, and LC480^®^ Probes Master Mix 2X for a final volume of 20 μl. The amplification program consisted of 5 min of incubation at 95°C, 45 cycles of 10 s at 95°C, 30 s at 60°C, and 1 s at 72°C, followed by a cooling step of 10 s at 40°C. Each plate contained one DNA extract from plasma considered as a control and a no template control. Validation of the amplification followed the sample rules for MCN. The deletion rate was obtained using the 2^–ΔΔCt^ method (42), and the result was normalized to a 10 ng DNA extract of plasma-rich platelets.

### Telomere Length Measurement

Telomere length was measured by a real-time PCR, using a technique described elsewhere (43). Succinctly, 10 ng of DNA samples was added to a mix of primers (final concentration of 900 nM, Supplementary Table 1) and LC480^®^ SYBR Green I Master Mix 2X (Roche, Germany) for a final volume of 30 μl. The following programs were applied: 10 min at 95°C, 40 cycles of amplification of 15 s at 95°C, 30 s at 60°C, and 30 s at 72°C. Each plate contained standards for telomere sequence ranging from 1.18 × 10^8^ to 1.18 × 10^4^ kb and for genomic sequence targeting, a single copy gene (namely, 36B4) ranging from 2.63 × 10^6^ to 2.63 × 10^2^ diploid genome copies. They also contained HEK293T DNA extracts and a well with no extract for the validation of sample amplification (44).

### Covariates

Excessive alcohol consumption was determined by the Alcohol Use Disorders Identification Test-Consumption (AUDIT-C) questionnaire, with a threshold greater than or equal to 4 for men and 3 for women (45). Poly substance use included any excessive alcohol consumption, daily heroin injection, or frequency of methamphetamine consumption greater than or equal to 4 times a month (38). Subjects were considered infected with HIV if HIV serology was positive and infected with HCV if HCV serology and HCV-viral load were positive. At the time of the sampling, most of the HIV-positive subjects were treated with antiretrovirals consisting of a combination of tenofovir/lamivudine/efavirenz (TDF/3TC/EFV) for the HIV infection, but none was treated for HCV.

### Statistical Analysis

Population characteristics are given as frequencies and percentages for categorical variables or mean values with standard deviation (SD) for numerical variables. A chi-squared test or *t*-test was performed for group comparisons (α = 5%). Raw data of TL/MCN/MDD were given with a median and confidence interval of 95% (CI 95%). For group comparison, TL, MCN, and MDD values were first log-transformed and then adjusted for age using linear regression. The difference between the group was assessed using the Wilcoxon signed *post hoc* test for the Tukey-adjusted least square mean (LSM) rank test (α = 5%).

In multivariate analysis, associations between psychiatric conditions (MDE and PS) and age-related biomarkers were assessed by binomial logistic regression, using MCN, MDD, and TL as continuous variables and adjusted for possible confounding factors (i.e., factors associated with both psychiatric disorder and aging-related biomarkers). Therefore, the selection of the variables was based on a literature review rather than a statistical criterion. These factors were related to heroin, methamphetamine, and alcohol consumption, which are risk factors for psychiatric disorders and have shown their potency to accelerate aging (37, 46, 47). We also tested HIV and HCV infections as they have been both linked with neuropsychiatric outcomes and accelerated aging (46, 48, 49). We also included ARV and methadone treatments, because they are able to induce immuno-inflammatory anomalies, which can accelerate premature cellular aging (50, 51) and direct inhibitory effect on enzymatic processes. At last, we included age and sex, which have both been linked with TL and mitochondrial dysfunctions: TLs are shortened with age and are longer in women than in men (52); mitochondrial dysfunction is accumulated with age (53). Altogether, the following variables were used: “age” (continuous), “sex” (men/women), “daily consumption of heroin” (yes/no), “frequency of methamphetamine consumption per month” (<4 times a month/ ≥4 times a month), “excessive alcohol consumption” (yes/no), “HIV serology” (positive/negative), “HCV infection” (positive/negative), “declare being on methadone assisted therapy” (yes/no), and “under ARV therapy” (yes/no). The number of variables included in each model followed the “one in ten” rule. No multiple testing correction was done. Final models were selected in a forward stepwise manner, using the smallest Akaike Information Criterion. Lastly, the models’ goodness of fit was assessed with Homer and Lemeshow test (α = 5%). The odds for any psychiatric condition depending on TL (continuous variable) were given for 0.1 unit change of TL, i.e., 0.1 log_10_ (kb/cell). Statistical analyses were performed on SAS^®^ studio university (Copyright© 2012–2020, SAS Institute Inc., Cary, NC, United States).

## Results

### Study Population and Characteristics

Overall, 299 subjects had a consultation with a psychiatrist between August 2018 and April 2019. In total, twenty subjects were excluded from the analysis because of incomplete data, and thirteen were also excluded because they had an isolated suicidal risk. In total, 130 subjects were diagnosed with at least one psychiatric condition and 136 had no diagnosis of psychiatric condition and were defined as our control population. A patient flowchart is available in Supplementary Figure 1. Out of the 130 PWIDs with a psychiatric condition, 110 (84.6%) were diagnosed with MDE, 74 (56.9%) with PS, and 54 (41.5%) were diagnosed with both (Table 1).

**TABLE 1:** Psychiatric diagnoses assessed by the MINI test, *n* (%).

Total of PWID with psychiatric condition		130 (100.0)
**Major depressive episode (MDE)**		
	MDE, total	110 (84.6)
	Current MDE only	52 (40.0)
	Past MDE only	19 (14.6)
	Current and past MDE	39 (30.0)
**Psychotic syndrome (PS)**		
	Current and/or past PS	74 (56.9)
**Patient with several psychiatric conditions**		
	MDE and PS	54 (41.5)

People who inject drugs with any psychiatric condition differed from PWIDs with no psychiatric condition. Notably, they were older, injected heroin for longer, smoked methamphetamine more often, used street methadone more frequently, had excessive alcohol consumption more often, and were less committed to their methadone program. However, the use of other drugs (cannabis, cocaine, amphetamine, ecstasy, and ketamine) was very limited and did not differ significantly between the two groups (data not shown). The prevalence rates of viral infections between the two groups were similar; more than two-thirds of HIV-infected participants were receiving first-line ARV treatment (Table 2).

**TABLE 2:** Baseline characteristics of the population.

	PWID with psychiatric conditions (*n* = 130)	Control population (*n* = 136)	*p*-value^1^
**Socio-demographic data**			
Sex, Male and transgender *n(%)*	120 (92.3)	127 (93.4)	0.73
Age, *mean(SD)*	45.3 (8.5)	40.9 (7.7)	<0.001
**Heroin, number of years practicing injection**			
<5 years	7 (5.4)	9 (6.6)	0.01
5 to 10 years	20 (15.4)	43 (31.6)	
10 to 15 years	36 (27.7)	34 (25.0)	
≥15 years	67 (51.5)	50 (36.8)	
**Heroin, frequency of injection during the month preceding the interview**			1.00
Less than once per day	43 (33.1)	45 (33.1)	
Daily	87 (66.9)	91 (66.9)	
**Methamphetamine, Positive urine test**	38 (29.2)	37 (27.2)	0.71
**Methamphetamine, number of years of consumption**			0.11
No consumption	28 (21.7)	45 (33.1)	
<2 years	26 (20.2)	19 (14.0)	
2 to 5 years	33 (25.6)	38 (27.9)	
≥5 years	42 (32.6)	34 (25.0)	
**Methamphetamine, frequency of consumption during the month preceding the interview**			0.73
<4 times a month	100 (76.9)	107 (78.7)	
≥4 times a month	30 (23.1)	29 (21.3)	
**Methadone (Positive urine test)**	71 (54.5)	85 (62.5)	0.19
**Excessive alcohol consumption**	45 (34.6)	32 (23.5)	0.05
**Poly substance use**			0.18
0	24 (18.5)	28 (20.6)	
1	58 (44.6)	72 (52.9)	
≥2	48 (36.9)	36 (26.5)	
**Viral infections**			
Positive HCV RNA	81 (62.3)	82 (60.3)	0.74
Positive HIV serology	47 (36.1)	57 (41.9)	0.34
HIV pos. receiving ARV treatment	33 (70.2)	43 (75.4)	0.26

*^1^Chi-squared test or t-test; HCV, hepatitis C; and HIV, human immunodeficiency virus.*

### Premature Aging Markers and Psychiatric Conditions

In total, 224, 256, and 253 samples had validated data for MCN, MDD, and TL, respectively. Reasons to invalidate the data were unmet quality control criteria and no blood sample available. TL was shorter after adjustment for age in PWIDs with an MDE (149.4 [139.9; 164.9]) and in PWIDs with both MDE and PS (154.2 [129.9; 162.7]), when compared to the control population. These differences were not significant (Table 3). TL was longer among PWIDs with PS (211.4 [147.9; 230.4] vs. 170.3 [160.8; 181.4], *p* = 0.01; Table 3). MCN values did not statistically differ between groups of PWID with MDE, PS, or both and the control group (Table 3). Finally, rate of MDD was higher in PWID with PS (0.55 [0.47; 0.69]) when compared to control group (0.72 [0.64; 0.80], but not statistically significant after adjustment for age, Table 3).

**TABLE 3:** Telomere length (TL), mtDNA copy number (MCN), and deletion rate (MDD) according to psychiatric condition, median [95%CI].

	Control population	Major depressive episode	Psychotic syndrome	Major depressive episode and psychotic syndrome
*n*	136	56	20	54
TL (kb/cell)	170.3 [160.8; 181.4]	149.4 [139.9; 164.9]	211.4 [147.9; 230.4]*	154.2 [129.9; 162.7]
MCN (c/cell)	438.5 [370.5; 497.1]	373.2 [280.8; 526.3]	278.8 [234.1; 506.4]	415.8 [321.4; 549.1]
MDD (%)	0.72 [0.64; 0.80]	0.71 [0.65; 0.78]	0.55 [0.47; 0.69]	0.77 [0.69; 0.89]

MCN, mitochondrial copy number; MDD, mitochondrial DNA deletion; TL, telomere length; and CI, confidence interval. Tested vs. control population (Wilcoxon signed-post hoc rank test for age-adjusted LSM); *p < 0.05. LSM, least square mean.

Additionally, PWID diagnosed with at least two episodes of MDE in their lifetime had shorter TL when compared to control group PWIDs after adjusting for age (*p* = 0.0025, respectively, Table 4). MCN and rate of MDD were not different according to the number of depression episodes (Table 4).

**TABLE 4:** Telomere length according to the multiplicity of the MDE, median [95%CI].

Multiplicity of episode	0 (control population)	1 episode	At least 2 episodes
*n*	136	71	39
TL (kb/cell)	170.3 [160.8; 181.4]	160.1 [151.1; 169.1]	129.9 [115.4; 144.4]*
MCN (cp/cell)	438.5 [372.9; 504.0]	404.8 [325.5; 484.0]	381.1 [236.5; 525.6]
MDD	0.72 [0.64; 0.79]	0.71 [0.63; 0.79]	0.79 [0.70; 0.88]

MCN, mitochondrial copy number; MDD, mitochondrial DNA deletion; TL, telomere length; and CI, confidence interval. Tested vs. control population (Wilcoxon signed-post hoc rank test for age-adjusted LSM); *p < 0.05. LSM, least square mean.

### Determinants of Psychiatric Conditions: A Multivariate Analysis

Because MDE and PS are two nosological different conditions, we grouped participants with MDE and PS regardless of their status for the other psychiatric conditions. Neither MCN nor MDD was associated with any psychiatric disorder in multivariate analyses when adjusted for confounders (data not shown). Regarding TL, we first found a strong interaction between psychiatric disorders, TL, and HCV infection. Therefore, we performed two analyses stratified on the subject of HCV status.

After stratification, the association between MDE and TL was strong for HCV-infected PWIDs, after adjustment for age, sex, daily heroin consumption, alcohol consumption, and not being on methadone treatment (adjusted odds ratio (aOR): 0.53 [0.36; 0.80]). Excessive alcohol consumption and not receiving methadone were associated risk factors of MDE (aOR: 3.23 [1.18; 8.87] and 3.03 [1.18; 7.80], respectively). Similar associations between PS and TL or PS and excessive alcohol consumption were observed among HCV-infected PWIDs, with aOR of 0.59 [0.39; 0.88] and 2.77 [1.37; 8.81], respectively, after adjustment for age, sex, and methadone treatment (Tables 5, 6). Among HCV-uninfected PWIDs, no significant association was observed between MDE and TL (aOR: 1.05 [0.72; 1.52]), after adjustment for age, methamphetamine consumption, and methadone treatment. PS was, however, associated with longer TL (aOR: 1.60 [1.01; 2.53]) and HIV-positive serology (aOR: 4.42 [1.20; 16.27]), after adjustment for age (Tables 5, 6).

**TABLE 5:** Determinants of “having a major depressive episode” among PWID.

	Major depressive episode					
	HCV positive (*n* = 161)			HCV negative (*n* = 105)		
	Univariate		Multivariate	Univariate		Multivariate
	OR	*p*-values	aOR [95%CI]	OR	*p*-values	aOR [95%CI]
**Telomere length *(continuous)***	**0.49 [0.34; 0.70]**	*<0.001*	**0.53 [0.36; 0.80]**	0.94 [0.66; 1.32]	*0.71*	1.05 [0.72; 1.52]
**Socio-demographic data:**						
Age (*continuous)*	**1.08 [1.03; 1.12]**	*<0.001*	**1.07 [1.01; 1.40]**	**1.05 [1.00; 1.10]**	*0.02*	**1.05 [1.00; 1.11]**
Sex (*ref : female*)						
Male and transgender	0.74 [0.14; 3.79]	*0.72*	0.21 [0.03; 1.51]	0.72 [0.22; 2.30]	*0.58*	–
**Drug consumption:**						
Heroin, frequency of consumption per month (*ref : less than daily*)						
Daily	0.79 [0.40; 1.55]	*0.49*	0.46 [0.17; 1.23]	1.04 [0.46; 2.34]	*0.92*	–
Methamphetamine, frequency of consumption per month (*ref:* <4 times per month)						
≥4 times per month	1.56 [0.73; 3.30]	*0.25*	–	**0.33 [0.11; 0.97]**	*0.03*	**0.27 [0.08; 0.94]**
**Alcohol (*ref: no excessive consumption*)**						
Excessive consumption	1.68 [0.81; 3.49]	*0.16*	**3.23 [1.18; 8.87]**	1.14 [0.51; 2.56]	*0.75*	–
**Viral infections:**						
HIV (*ref: HIV serology negative*)						
HIV serology positive	0.65 [0.34; 1.21]	*0.17*	–	1.15 [0.45; 2.91]	*0.76*	–
**Treatments:**						
**Methadone (*ref: currently receiving methadone*)**						
Not receiving methadone	1.51 [0.80; 2.84]	*0.20*	**3.03 [1.18; 7.80]**	**2.56 [1.10; 5.98]**	*0.03*	2.44 [0.90; 6.84]
ARV (*ref: no*)						
Under ARV	0.69 [0.36; 1.34]	*0.28*	–	1.11 [0.39; 3.20]	*0.84*	–

*ref, non-syndrome; aOR, adjusted odd ratio; and CI, confidence interval. Values in bold are statistically significant.*

**TABLE 6:** Determinants of “having a psychotic syndrome” among PWID.

	**Psychotic syndrome**					
	HCV positive (*n* = 161)			HCV negative (*n* = 105)		
	Univariate		Multivariate	Univariate		Multivariate
	OR	*p*-values	aOR [95%CI]	OR	*p*-values	aOR [95%CI]
**Telomere length *(continuous)***	**0.65 [0.46; 0.92]**	*0.02*	**0.59 [0.39; 0.88]**	1.45 [0.98; 2.16]	*0.06*	**1.60 [1.01; 2.53]**
**Socio-demographic data:**						
Age (*continuous)*	1.03 [0.98; 1.07]	*0.23*	0.98 [0.93; 1.04]	1.04 [0.99; 1.09]	*0.10*	1.10 [1.03; 1.18]
Sex (*ref : female*)						
Male and transgender	0.79 [0.14; 4.48]	*0.79*	0.39 [0.05; 2.77]	2.17 [0.45; 10.4]	*0.30*	–
**Drug consumption:**						
**Heroin, frequency of consumption per month (*ref: less than daily*)**						
Daily	0.75 [0.36; 1.56]	*0.45*	–	1.35 [0.54; 3.37]	*0.52*	–
Methamphetamine, frequency of consumption per month (*ref:* < 4 times per month)						
≥4 times per month	**2.30 [1.05; 5.03]**	*0.04*	–	0.66 [0.22; 1.99]	*0.45*	–
**Alcohol (*ref: no excessive consumption*)**						
Excessive consumption	**2.62 [1.22; 5.63]**	*0.01*	**2.77 [1.37; 8.81]**	1.13 [0.46; 2.75]	*0.78*	–
**Viral infections:**						
**HIV (*ref: HIV serology negative*)**						
HIV serology positive	0.55 [0.27; 1.10]	*0.09*	–	**3.18 [1.21; 8.36]**	*0.02*	**4.42 [1.20; 16.27]**
**Treatments:**						
**Methadone (*ref: currently receiving methadone*)**						
Not receiving methadone	2.02 [0.98; 4.19]	*0.05*	1.94 [0.83; 4.50]	1.58 [0.63; 3.94]	*0.32*	–
**ARV (*ref: no*)**						
Under ARV	0.51 [0.24; 1.09]	*0.07*	–	2.23 [0.76; 6.60]	*0.15*	–

*ref, non-syndrome; aOR, adjusted odd ratio; and CI, confidence interval.*

*Values in bold are statistically significant.*

## Discussion

Herein, we reported that among the canonical age-related biomarkers, telomere attrition was associated with mental depression episodes and PSs in HCV-infected PWID, while mitochondrial genotoxicity markers were not. The risk of MDE was increased approximately two-fold in HCV-infected PWIDs when total leukocyte TL was reduced by approximately 10% of its baseline value when compared with PWID without any psychiatric condition. Meanwhile, such an association was not observed among HCV-uninfected subjects. Given a conservative approach to fix a 59 bp telomere loss per chromosome and per year of life (54), the telomere age of the PWID with one MDE can be considered 4.2 years older than the PWIDs with no MDE, and for those with a two and more MDEs 12.8 years older. TL attrition is related to accelerated biological aging and therefore a risk factor for age-related diseases, such as cardiovascular diseases, diabetes, atherosclerosis, neurodegenerative diseases, cancer. Overall, TL attrition increased the risk of frailty and mortality (11, 55).

Telomere length attrition was one of the earliest described markers of premature aging, often a consequence of low-grade inflammation and oxidative stress (56). Yet, in neuropsychiatric diseases, many studies have evidenced immune dysfunctions, such as increased systemic and brain pro-inflammatory cytokine, increased C-reactive protein concentrations in peripheral blood, and the infiltration of peripheral immune cells through the blood-brain barrier, microglial activation, and increased peripheral innate immunity activity (57, 58). Therefore, mental disorders are associated with an increase in oxidative stress and impairment of immuno-inflammation homeostasis.

Many studies have previously analyzed the link between TL and mental disorders in adults who do not use drugs. Depression, in particular, has been extensively investigated. Nearly all studies on the subject matter have found shorter TL to be associated with MDE (22, 59–62), with few exceptions (63). Meanwhile, PS and schizophrenia have been studied less often and have produced more heterogeneous results: finding decrease (61, 64, 65), increase (32, 66), or no change (67, 68) in TL when compared to the control group. However, two meta-analyses found a slight association between shorter TL and schizophrenia (65, 69). More recently, a meta-analysis that includes 14,827 subjects confirmed that shorter TL was associated with any psychiatric disorder; the association with depression remained significant when subgroup analyses were performed, while the association with PS was no longer significant (70). Herein, we reported the same association among HCV-infected PWIDs but with additional aggravating factors, such as multiplicity of depression episodes. Furthermore, the multivariate analysis showed excessive alcohol consumption as a risk factor for mental disorders as previously reported (71, 72). Mental disorders are listed among the extra-hepatic symptoms of HCV infection (73). Recently, Yao’s group demonstrated that circulating CD4 + T cells from patients who are chronically HCV infected exhibit an immune activation status that leads to telomere damage (74). Heroin use has been related to immune impairment in the brain and increased neuroinflammatory response; moreover, studies have revealed TL attrition and decreased telomerase activity in the blood of heroin users when compared to controls (37, 75, 76). Methamphetamine use also showed an increase in oxidative stress and inflammation in the brain and has recently been associated with a shorter TL among HIV-positive subjects (46, 77). Ultimately, PWIDs belong to a population that is poly exposed to multiple risk factors that possibly play a role in the underlying and damaging processes that drive telomere shortening and premature biological aging.

Mitochondrial dysfunctions assessed through mtDNA alterations have also been proposed as markers for premature aging. Similarly, TL erosion, oxidative stress, and inflammation increase mtDNA damage and lead to the loss and impairment of mtDNA copies (78). Previous reports have shown inconsistent results in terms of MCN in blood between depressed and controls. The same trends were reported for cell-free circulating MCN (ccf-MCN) in plasma, which has been proposed as a more accurate biomarker for mitochondrial disruption, with decreased (79) or increased (24) ccf-MCN. Only a few studies have looked for MCN in the blood of schizophrenic patients; they have found a slight decrease in MCN among patients when compared to the controls (80–82); although no difference in ccf-MCN was observed (79). Studies on mtDNA integrity, i.e., point mutation or deletion, are scant but the general trend shows increased mtDNA damage among patients with psychiatric disorders (25, 27). In line with these disparate observations, we did not find a significant difference in MCN, neither among PWIDs suffering from MDE nor among PWIDs suffering from PS. Additionally, we did not find any difference in MDD between the two populations. To summarize, mtDNA alteration as a biomarker for mental health in PWID is not straightforward, as they are for non-drug users.

This study has limitations. First, the MINI used in this study did not diagnose anxiety conditions, which are highly prevalent among PWIDs and which have been previously associated with premature aging (83). Second, we did not have a group of non-drug users with or without mental disorders that would have been useful in positioning our findings in a broader context. Thirdly, aging markers were analyzed in whole blood DNA extracts. Conclusions cannot be extrapolated to subsets of lymphocytes known to have reduced TL upon HIV infection and/or ARV exposure (84, 85). Fourth, the mtDNA integrity did not encompass the point mutation rate, as it required sequencing techniques. Finally, the participants were recruited outside the healthcare system and they did undergo clinical examination. Consequently, comorbidities and other polymedications were not recorded and could not be used as confounding variables in the various analyses.

## Conclusion

Overall, we reported TL attrition among HCV-infected PWIDs suffering from mental disorders, a marker for premature biological aging. We, therefore, suggest that this subset of PWIDs with mental disorders is at higher risk of age-related diseases because of premature TL attrition rather than mtDNA alterations, particularly among those who are suffering from depression.

## Data Availability Statement

The raw data supporting the conclusions of this article will be made available by the authors, without undue reservation.

## Ethics Statement

The studies involving human participants were reviewed and approved by Scientific Advisory Board of DRIVE (NCT03526939) Haiphong University of Medicine and Pharmacy’s Ethics Review Board. The patients/participants provided their written informed consent to participate in this study.

## Author Contributions

NN, LM, PV, and J-PM: conceptualization. NN and PM: methodology. MD and AV: formal analysis. SL, RV, and J-PM: validation. HD, DR, HG, CQ, and NT: investigation. MD and J-PM: data curation, and writing—original draft preparation. NN, LM, VV, JF, PV, DD, and DL: writing—review and editing. HD, KH, DD, and NN: funding acquisition. All authors have read and agreed to the published version of the manuscript.

## Conflict of Interest

The authors declare that the research was conducted in the absence of any commercial or financial relationships that could be construed as a potential conflict of interest.

## Publisher’s Note

All claims expressed in this article are solely those of the authors and do not necessarily represent those of their affiliated organizations, or those of the publisher, the editors and the reviewers. Any product that may be evaluated in this article, or claim that may be made by its manufacturer, is not guaranteed or endorsed by the publisher.
