# Supplementary material for: Mental Disorders Are Associated With Leukocytes Telomere Shortening Among People Who Inject Drugs
Source: Front Psychiatry. 2022 Jun 17;13:846844. doi: 10.3389/fpsyt.2022.846844 (PMC9247253; doi:10.3389/fpsyt.2022.846844)
Supplement: Supplementary file 1 [file Data_Sheet_1.PDF]

Supplementary tables and figures

Supplementary figure 1: Flowchart of population of study.

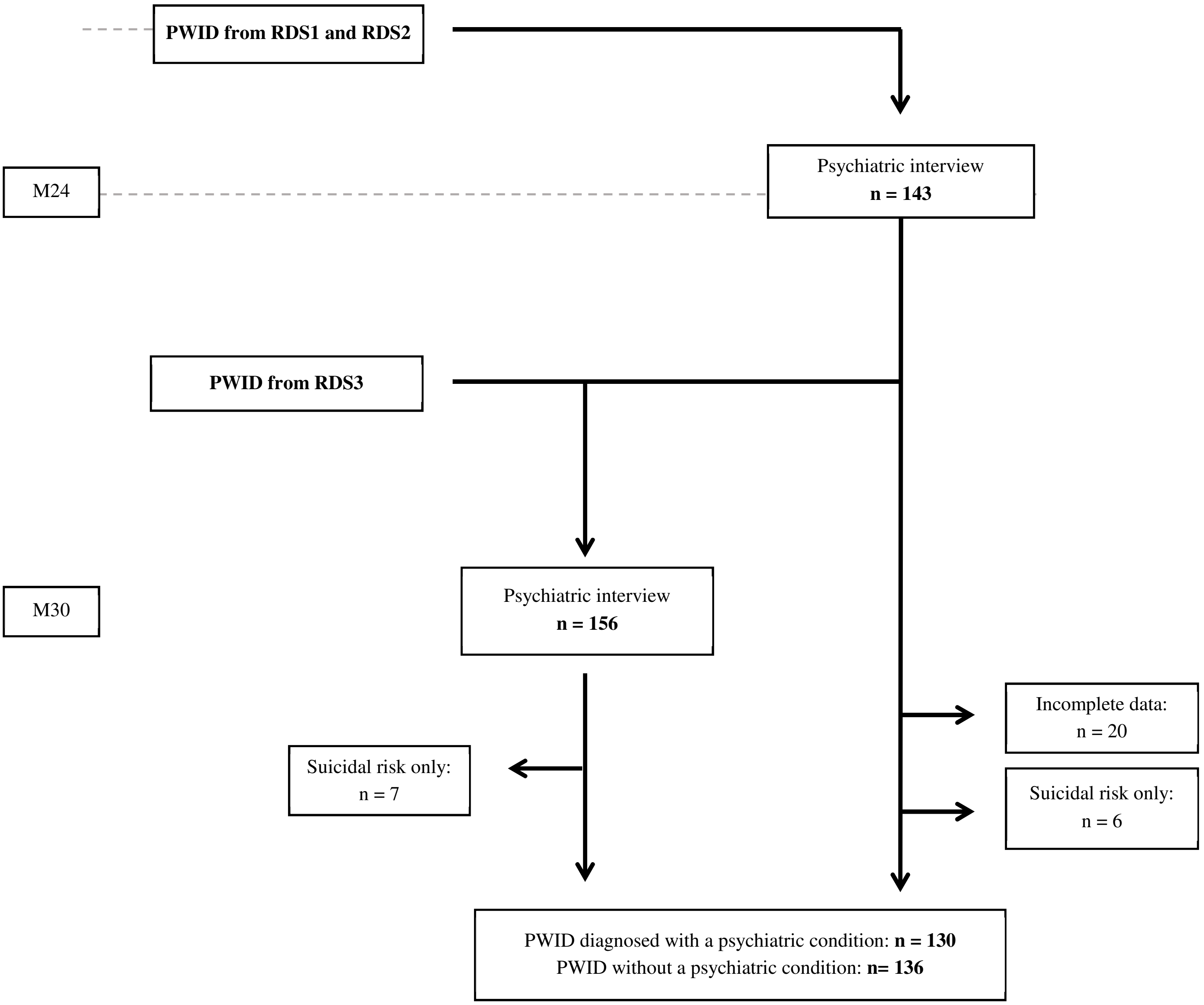

Supplementary table 1: Primers and probes for mtDNA and telomere length assays.

Names	*Sequence*	*Length (bp)*
**mtDNA copy number (MCN)**		
U1A - *forward*	5′-TGCGCCTCTTTCTGGGTGTT-3′	20
U1A - *reverse*	5′-CGGCATGTGGTGCATAA-3′	17
mtDNA - *forward*	5′-GTAATCCAGGTCGGTTTCTA-3′	20
mtDNA - *reverse*	5′-GGGCTCTGCCATCTTAA-3′	17
**mtDNA deletion (MDD)**		
ND1 - *forward*	5′-CCCTAAAACCCGCCACATCT-3′	20
ND1 - *reverse*	5′-GAGCGATGGTGAGAGCTAAGGT-3′	22
ND1 - *probe*	5′-FAM-CCATCACCCTCTACATCACCGCCC-TAM-3′	24
ND4 - *forward*	5′-CCATTCTCCTCCTATCCCTCAAC-3′	23
ND4 - *reverse*	5′-ACAATCTGATGTTTTGGTTAAACTATATTT-3′	30
ND4 - *probe*	5′-HEX-CCGACATCATTACCGGGTTTTCCTCTTG-BHQ1-3′	28
**Telomere length**		
36B4 - *forward*	5′-CCCATTCTATCATCAACGGGTACAA-3′	25
36B4 - *reverse*	5′-CAGCAAGTGGGAAGGTGTAATCC-3′	23
Telomere - *forward*	5′-CGGTTTGTTTGGGTTTGGGTTTGGGTTTGGGTTTGGGTT-3′	39
Telomere - *reverse*	5′-GGCTTGCCTTACCCTTACCCTTACCCTTACCCTTACCCT-3′	39
